# Clinical management of resistance evolution in a bacterial infection: A case study

**DOI:** 10.1093/emph/eov025

**Published:** 2015-10-10

**Authors:** Robert J. Woods, Andrew F. Read

**Affiliations:** ^1^Division of Infectious Diseases, Department of Internal Medicine, University of Michigan, Ann Arbor, MI 48109, USA; ^2^Department of Biology, Center for Infectious Disease Dynamics, Pennsylvania State University, University Park PA 16802, USA; ^3^Department of Entomology, Center for Infectious Disease Dynamics, Pennsylvania State University, University Park PA 16802, USA

**Keywords:** antibiotic resistance, resistance management, evolutionary risk, clinical decisions, *Enterobacter*, MRSA

## Abstract

We report the case of a patient with a chronic bacterial infection that could not be cured. Drug treatment became progressively less effective due to antibiotic resistance, and the patient died, in effect from overwhelming evolution. Even though the evolution of drug resistance was recognized as a major threat, and the fundamentals of drug resistance evolution are well understood, it was impossible to make evidence-based decisions about the evolutionary risks associated with the various treatment options. We present this case to illustrate the urgent need for translational research in the evolutionary medicine of antibiotic resistance.

## Abstract (precis)

This chronic bacterial infection evolved extensive resistance, killing the patient. Evolutionary science is insufficiently developed to better manage such life-threatening evolution.

## INTRODUCTION

Antibiotic therapy represents one of greatest achievements of modern medicine, but this achievement is threatened by the growing challenge of antibiotic resistance. A case is described of a patient with a chronic, open bacterial infection for which definitive source control could not be obtained. The patient eventually died because her infection evolved resistance to available antibiotics. This patient’s presentation is characteristic of a broad array of cases where the primary determinant of the quality and quantity of life is the rate of resistance evolution. Such patients may also be a source of resistant ‘superbugs’ in health care settings. Our goal is to describe the nature of the clinical choices that had to be made and the knowledge deficits that prevented the application of evolutionary principles in this clinical setting. By more clearly defining the problem we hope to encourage the search for solutions.

## CASE PRESENTATION

**Figure 1.**
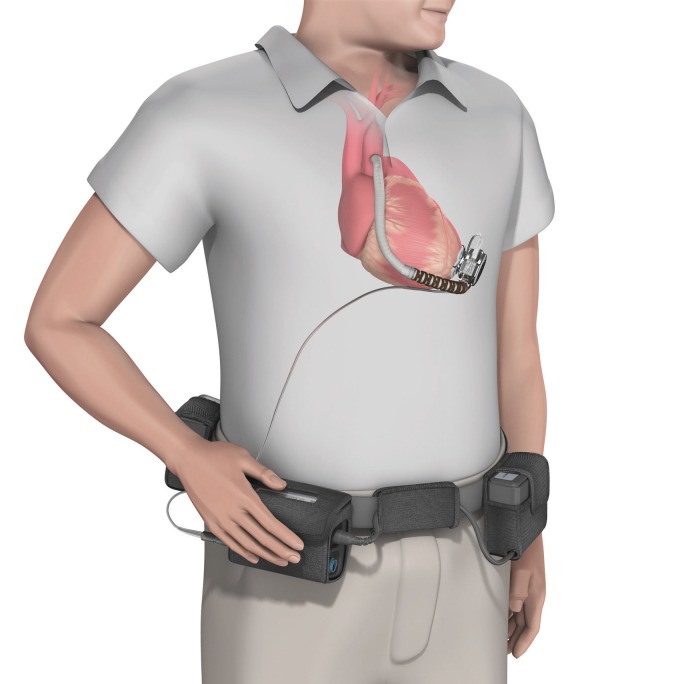
Schematic of a left ventricular assist device (LVAD) *in situ*, with driveline to external power source. CT scans of the patient revealed evidence of bacterial build up around the efferent limb of the LVAD and the anastamosis to the ascending aorta. The presumed route of invasion was the drive line. Reproduced from http://www.heartware.com/media-resources with permission.

The patient was a 56-year-old female with a medical history of type 2 diabetes, hyperlipidemia, pulmonary embolism and nonischemic cardiomyopathy diagnosed 15 years prior. The cardiomyopathy was complicated by secondary pulmonary hypertension and paroxysmal atrial fibrillation. An implantable cardioverter-defibrillator was placed for primary prophylaxis of sudden cardiac death. She underwent Heartware® Left Ventricular Assist Device (LVAD) placement 2 years prior to presentation. She was not a candidate for heart transplant. She presented with a small amount of brown-colored discharged from around the LVAD driveline, which connects the LVAD to the external power source (Fig. 1). She had no fevers, chills, night sweats or weight loss. Her dyspnea was stable, and she had no chest pain and no pain along the driveline. On exam, she was an obese female who was in no acute distress. She was afebrile, with regular heart rate and baseline blood pressure. Inspection of the driveline entry site showed a scant amount of bloody drainage. Laboratory values were notable for a normal white blood cell count and creatinine. The drainage from the driveline was cultured and grew methicillin resistant *Staphylococcus aureus* (MRSA), which was resistant to tetracycline but sensitive to trimethoprim/sulfamethoxazole (Bactrim), erythromycin, clindamycin and vancomycin. An ultrasound was performed that showed no fluid pocket along the driveline. Treatment with vancomycin was initiated via a peripherally inserted central catheter (or PICC line), and she was discharged from the hospital.

## CASE MANAGEMENT AND OUTCOME

**Figure 2.**
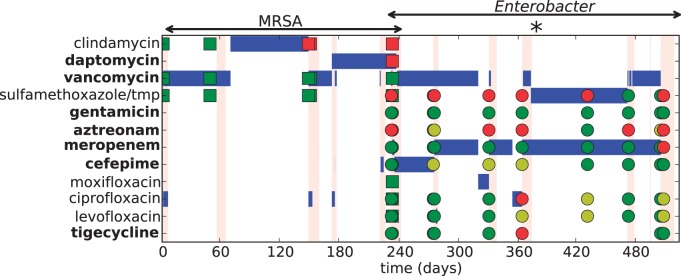
The patient’s course from initial signs of LVAD driveline infection through death. Bars in blue show administration of drug, vertical pink lines the timing and duration of hospital visits and the symbols show levels of resistance to the various antibiotics of MRSA (squares) and *Enterobacter* (circles) isolates taken at various time points, where green is defined as susceptible, yellow is intermediate susceptibilities, and red is resistance. MIC were measured with Vitek or E-test and cutoffs were standard CLSI break points. Drug names in bold can only be given intravenously for this infection. Asterisk shows the timing of the clinical decision discussed in the main text

The timing of subsequent hospital visits, antibiotic treatments and the resistance profiles of bacteria isolated from the patient are summarized in Figure 2. The patient had monthly scheduled appointments at an infectious disease outpatient clinic. Throughout, resistance phenotypes were defined as sensitive, intermediate or resistant based on Clinical and Laboratory Standards Institute (CLSI) breakpoints with minimal inhibitory concentrations (MIC) measured by Vitek or e-test.

The patient was seen in clinic 4 weeks after the original presentation. She continued to be without significant symptoms other than persistent drainage around the driveline, indicating on-going infection. Culture of the drainage again grew MRSA, with the same resistance pattern. Vancomycin was continued. When she returned to clinic at 8 weeks, the drainage was persistent. The PICC line was removed to limit the risk of a PICC line-associated infection, and the patient started clindamycin orally. She remained on clindamycin for about 4 months, when she presented to the hospital with fevers, elevated white blood cell count and left-sided abdominal pain. She was found to have bacteremia with MRSA that now was resistant to clindamycin. Ultrasound of the driveline showed no fluid accumulation amenable to surgical drainage. Vancomycin was restarted, and she left the hospital.

She returned to the hospital 1 month later with confusion and increasing abdominal pain around the driveline. One of two blood cultures grew a coagulase negative *Staphylococcus* (considered to be a contaminant). Urine culture grew vancomycin resistant *Enterococcus* (VRE). She was discharged from the hospital on daptomycin via a PICC line, to cover the MRSA and the VRE. Persistent drainage from the driveline was noted. She presented again 2 months later with fever, leukocytosis and acute renal injury. Blood cultures were negative. Ultrasound of the driveline showed no pocket of fluid accumulation. A urine culture grew an extended spectrum beta-lactamase (ESBL) producing *Escherichia coli*. Broad-spectrum antibiotics were used throughout the hospitalization (daptomycin and cefepime, and briefly vancomycin) and she was discharged on daptomycin alone.

One month later she presented to the hospital with fever. Computed Tomography (CT) scan of the chest revealed gas bubbles around the LVAD and the anastomosis to the ascending aorta (Fig. 1). Incision and drainage of the abscess pocket was performed and the wound was left open to allow daily dressing changes. Blood cultures grew *Enterobacter cloacae*, and cultures from the abscess grew *Enterobacter* and MRSA. The MRSA had a daptomycin MIC of four (resistant). Daptomycin was discontinued, and vancomycin was restarted. The patient was discharged on cefepime and vancomycin.

Two months later she was admitted with fatigue, fever and elevated white blood cell count. Blood and wound cultures again grew *Enterobacter*, but the cefepime MIC had increased from 1 and 2 (sensitive) to 8 (intermediate resistance). Cefepime was stopped and meropenem was started. CT imaging was stable, and did not reveal a drainable abscess. She was seen again in clinic after 6 weeks of meropenem and vancomycin. The wound was still open, and was being packed daily, but appeared to be improving. The PICC line was removed, and she was started on moxifloxacin (to cover both infections). Twelve days after switching to moxifloxacin she was admitted with fatigue and abdominal pain. Blood cultures grew *Enterobacter*. The patient was switched back to meropenem, requiring a PICC. No coverage for the MRSA was used at that time.

The patient was seen in clinic 6 weeks later, nearly 1 year from her initial presentation, at which point the PICC line was removed and the patient started high dose ciprofloxacin, to cover both MRSA and Enterobacter. The patient was admitted three days later (Fig. 2, asterisk) with fevers, chills and shortness of breath. Blood cultures were again positive for *Enterobacter*, now resistant to ciprofloxacin. Meropenem was restarted. The patient was discharged on meropenem for the *Enterobacter* and Bactrim for the MRSA.

The patient remained on Bactrim and meropenem for 4 months when she was admitted to the hospital with fatigue, subjective fevers, ‘shakiness’ and elevated white blood cell count. One out of two blood cultures on admission were positive for *Corynebacterium* that was believed to be a contaminant. The Bactrim was discontinued and vancomycin started. She was discharged after a week when her energy subjectively improved, although her leukocyte count remained elevated. The patient was readmitted to the hospital 3 weeks later with fatigue, and she was discharged the subsequent day on the same medications. Blood cultures were negative.

Three weeks later, she presented again with progressive lethargy. Blood culture grew *Enterobacter* with a meropenem MIC of 4 (sensitive), and the meropenem was continued, but 3 days into the hospitalization the blood cultures grew *Enterobacter* with a meropenem MIC of >16 (resistant). The antibiotic was switched to a combination of cefepime and vancomycin (this *Enterobacter* had a cefepime MIC of ≤1, sensitive). However the patient became increasingly obtunded, the white blood cell count continued to rise. The patient’s family decided to withdraw care. She succumbed to the infection.

## DISCUSSION

Infection involving prosthetic material, such as an LVAD, is virtually impossible to eradicate without removal of the device, which could not be done in this case. While this patient’s initial infection was susceptible to multiple antibiotics, successive antibiotic choices were met with increasing antibiotic resistance (Fig. 2). The evolution of antibiotic resistance became the key threat to the clinical outcomes that mattered most: the duration and quality of her remaining life. However, clinically relevant data to guide treatment choices to minimize resistance evolution are remarkably limited.

The time to clinical failure of a treatment regimen due to resistance evolution is determined by the duration of two phases [1].The first phase is the time taken for a resistant organism to first appear in the infection. Resistance arises by mutation, horizontal gene transfer (HGT) or immigration of already resistant bacteria. The second phase is the time taken for resistant organisms to replicate and spread to life-threatening densities. A fundamental dilemma is that many treatment choices have contrasting impacts on the duration of these two phases [2].

### *Clinical Decision-Making*

The goal of treatment was to identify a regimen that gave an immediate improvement in the patient’s health and at the same time maximally delayed the emergence of resistance. Decisions had to be made concerning choice of drug(s), dose, infusion time and dosing frequency and about whether to maintain a regimen until it failed and then switch to another, or whether to change treatment after some fixed interval, perhaps rotating treatments.

To illustrate the issues involved, we focus only on the choice of drug(s) and consider the options available for the treatment of the *Enterobacter* infection when the patient was admitted to the hospital 1 year into the infection (Fig. 2, asterisk). At that point, it was felt that antibiotic treatment could not be stopped because doing so would result in the infection rapidly overwhelming the patient.

Based on the resistance profiles of the *Enterobacter* isolated from blood and wound cultures, the drug options were (i) continuing a carbapenem such as meropenem, (ii) switching back to cefepime, a fourth generation cephalosporin, to which the *Enterobacter* was not fully resistant, (iii) combination therapy with meropenem and cefepime, which is not typically considered an option clinically due to lack of data, (iv) combining one of those antibiotics with another agent for which there was resistance, such as a fluoroquinolone like levofloxacin and/or (v) treatment for a short duration with an antibiotic with significant side effects that would limit prolonged use such as aminoglycoside (e.g. gentamicin) or colistin.

None of these treatment options is clearly superior based on immediate health outcomes in the treatment of *Enterobacter*. A small case series with 12 *Enterobacter* infections shows that 3 failing cefepime therapy were successfully treated with a carbapenem [3]. However, in a series of 51 patients with *Enterobacter cloacae* bacteremia, cefepime and meropenem therapy had similar outcomes [4]. In a propensity-matched cohort study, 32 patients treated with cefepime had outcomes similar to 32 patients treated with meropenem for bacteremia, pulmonary infection or intra-abdominal infections [5]. These studies are of tenuous relevance to situation of a chronic LVAD infection in which source control cannot be obtained.

Moreover, those studies say little about evolution, a key issue in this case. A major question was whether sequential monotherapy, e.g. meropenem until failure then cefepime or vice versa (options 1 and 2 above), would select for cross-resistance more or less rapidly than would combination therapy with two drugs (option 3). Answering that question involves estimating the impact of sequential and combination therapy on both the origin and the spread of meropenem resistance, cefepime resistance and meropenem–cefepime cross resistance. Combination therapy can reduce the probability of resistance mutations arising because the probability of multiple resistance mechanisms arising *de novo* in the same bacterium can be vanishingly small, so long as the chosen drugs have independent mechanisms of resistance [1]. However, both antibiotics are beta-lactams. There are resistance mechanisms unique to each drug and mechanisms that confer resistance to both. Alternatively, meropenem could be combined with one of the earlier-used drugs, like ciprofloxacin against which resistance had already arisen (option 4), in the hope that the cost of resistance to ciprofloxacin would, in the presence of meropenem, favor ciprofloxacin-sensitive bugs [1, 6, 7]. However, combination therapy may also lead to more rapid emergence of a fully resistant organism by way of acquisition of multi-drug resistance genes such as those encoding efflux pumps, or by acquisition of mobile genetic elements that carry resistance genes to multiple classes of antibiotics. It is not clear if adding an additional drug for brief periods to either mono- or combination therapy (option 5) would help retard resistance evolution.

A large literature on these drugs and *Enterobacter* and related bacteria shows that each of the possible routes of resistance evolution can occur (chromosomal mutations, cross resistance, plasmids, immigration of resistant bacteria). For example, a common step in *de novo* resistance evolution for both cefepime and meropenem is often de-repression of the chromosomally encoded ampC beta-lactamase [8, 9] but this derepression by itself is unlikely to yield clinical resistance to either drug [10, 11]. Cefepime resistance may also require mutations in ampC that improve catalytic activity [12, 13], which have also been observed in other members of this bacterial genus and family [14, 15]. For meropenem, ampC mutations plus outer membrane mutations proteins (ompF) may be required for clinical resistance in *Enterobacter* [16] and *Serratia* [17]. The evolution of carbapenem resistance *in vivo* has been seen to involve both ampC induction and mutations that reduce permeability [18], or may occur in the absence detectable beta-lactamase activity [19]. The risk of acquiring antibiotic resistance through horizontal gene transfer introduces even more uncertainty. Plasmid-borne beta-lactamases often, but not always, confer resistance to the multiple betalactams (reviewed by [20]), and frequently carry resistance to other classes of antibiotics [21, 22]. Furthermore, antibiotic use and indwelling catheter, as was used in this patient, are risk factors for colonization and infection with carbapenem-resistant organisms [23, 24].

Thus, each of the possible routes of resistance evolution can occur, but knowing that something can occur says very little about the likelihood that it will, and the likelihoods are what matter. It should be possible to estimate those probabilities. For instance, what were the chances that this patient’s *Enterobacter* infection would acquire plasmids conferring cross-resistance? That depends on the likelihood such plasmids exist in her microbiota, her home environment, the hospital, the local region—and whether bacteria bearing such plasmids will contact her *Enterobacter*. Likewise, if chromosomal mutations are the primary source of resistance, how likely were mutations conferring cross-resistance? If the probability of acquiring plasmids or mutations which confer cross-resistance is low, successive monotherapy (meropenem then cefepime) will likely fail faster than combination therapy (both together). What was the probability of new resistant pathogens invading? Even targeted treatment can create resistance elsewhere in a patient’s microbiome. What was the probability that off-target evolution in non-pathogenic species would be a significant source of resistance for the *Enterobacter* infection or of new resistant bacteria species?

The general topic of combination therapy against bacteria has a long, and somewhat controversial history in clinical medicine [25–29]. Combination therapy to prevent resistance emergence was shown in early head-to-head trials in tuberculosis [30, 31]. This logic cannot be directly extended to our case. Large meta-analyses of Gram negative bacteremia have not found general support for improved outcomes with combination therapy [27, 28]. A separate meta-analysis specifically looking at the evolution of resistance similarly saw no difference between use of a beta-lactam compared to a beta-lactam plus an aminoglycoside [32]. A large meta-analysis with 173 drug trials, which looked at a broad collection of bacterial infections and antibiotics, did not show a significant difference in resistance emergence between combination therapy and monotherapy, with the exception of penicillin and aminoglycoside monotherapy which has a slightly higher rate [33]. While patients in such trials are not directly analogous to the chronic LVAD infection presented here, they caution against extrapolating from TB.

Clinicians at a patient’s bedside must weigh treatment options with respect to ultimate outcomes. Despite a comprehensive search of the literature, we concluded that it was impossible to make even crude estimates of the evolutionary risks associated with the different treatment options. Consequently, we decided on a treatment plan that avoided potential toxicity, and administered the drug in a manner that was easiest for the patient. Thus meropenem, the strongest single drug against *Enterobacter,* was used as monotherapy. Dosing was 1 g every 8 h, easier than the alternative 500 mg every 6 hours. The drug choice was based on the earlier hints of lowered susceptibility to cefepime and to keep a drug available should meropenem fail. After meropenem resistance arose, the patient rapidly succumbed to infection, despite being switched to cefepime.

### *Future Research Needs*

In Box 1, we list specific questions that, had we been able to answer them, would have led to better clinical outcomes. They can all be addressed with currently available technologies. For example, *post hoc* whole-genome studies of longitudinal isolates taken from patients can directly address how resistance evolved following a given set of choices. Microbiome approaches can define the community dynamics and the potential donor pool of resistance genes. The likelihood of acquiring cross-resistance by HGT could be estimated by examining local and regional isolate collections for cross-resistance. Eventually trials could be done to test which antibiotic choices improve evolutionary outcomes. It remains to be determined whether general rules of thumb can be developed to guide decision making (e.g. start with combination therapy from the beginning, or always use the least amount of the narrowest spectrum drug), or whether each situation will require a different approach (personalized medicine). There is the very interesting possibility that experimental evolution done in real time with bacteria taken from a patient could inform clinical decisions and lead to improved patient outcomes (diagnostic experimental evolution). How does evolutionary risk depend on the pathogen, the patient, the infection and the care setting?

Box 1. Tractable Research Questions

Treatment strategies in this case could not be designed to slow the evolution of resistance, because data upon which to develop such a strategy were unavailable. Answers to the following questions would have enabled evidence-based clinical decision-making about the evolutionary risks associated with the various treatment options.

- Does resistance in *Enterobacter* infections more likely arise from *de novo* mutations or genetic elements acquired from other bacteria?
- How readily can cross-resistance to meropenem and cefepime evolve by chromosomal mutation? How many mutations are involved compared with resistance to monotherapy?
- What is the probability that the *Enterobacter* infection could acquire plasmids conferring resistance to all beta-lactams, fluoroquinolones or aminoglycosides?
- Ciprofloaxcin was abandoned after some resistance was detected. Had meropenem been used in combination with ciprofloxacin, was it is more likely that ciprofloxacin sensitivity would return, or that ciprofloxacin–meropenem cross-resistance would arise?
- Long-term Bactrim use was met with increasing Bactrim sensitivity in the *Enterobacter*. Why? Did dual therapy (Bactrim and meropenem) select for increasing Bactrim sensitivity?
- Did the switch from Bactrim to vancomycin (which was done for the MRSA) change the outcome of *Enterobacter* evolution?
- How would the available treatment options shape the patient’s microbiome, and how does that shape the risk of HGT or a secondary infection with a highly resistant organism?
- Would the addition of short duration treatment with an aminoglycoside or colistin have prevented the emergence of meropenem resistance?
- Once the *Entrobacter* infection was established, the MRSA infection was not seen. Did interspecific competition drive the MRSA to extinction (or at least irrelevance)?
- The patient harbored other infections, including an ESBL producing *E. coli* and VRE in the urine. Should this knowledge play into the antibiotic choices?
- Where was the source population that was generating blood stream infections? Was this the biofilm on the device? Did the size of that population relate to the probability of blood stream infection? If yes, how to balance the immediate clinical benefit of reducing that source population without imposing maximum selection for resistance?
- How could the infection have been in contact with other bacteria? How likely was such cross-talk?
- Could more effective decisions be made if a larger sample of bacteria was phenotyped for resistance? If rare meropenem resistance was discovered, could reducing the meropenem dose reduce competitive release and hence prolong the clinical effectiveness of meropenem? Or would it be more effective to switch drug immediately, allowing competition to eliminate the resistance while it is still rare?
- Once resistance is identified should that drug be stopped? Or is there a continued role to play, (antagonisms between resistance mechanisms, fitness cost of resistance, potential for reversion to sensitivity in presence of combination therapy)?
- Should drug swaps be guided by resistance profiles, or done before resistance is detected clinically?
- What happened in the last 3 days? Why did resistance evolve?
- When would a minimalist antibiotic approach slow resistance evolution, and when might it promote resistance evolution?

### Conclusion

We contend that empirically informed evolutionary risk management of chronic bacterial infections should be possible. There is precedence for this in HIV infections, where sequencing of viral isolates in real-time directly informs choice of drug combination [34]. Combination therapy is the standard of care for HIV because single drug and two drug combinations result in resistance evolution, but three drug combinations result in sustained suppression [35–37].That strategy of evolutionary management has made HIV a survivable infection, a success story largely arrived at empirically by physicians. It remains to be seen whether an analogous solution can be achieved for chronic bacterial infections, and whether evolutionary science can help. In our view, the fundamental factors determining the speed of resistance evolution are relatively well understood from mathematical, in vitro and animal models [e.g. 1, 2]. However, turning that fundamental knowledge into useful estimates of the evolutionary risks associated with various clinical options is a big step, and one that has largely not been taken for chronic bacterial infections like those that blighted this patient. A real test of fundamental evolutionary science is whether it can be used to predict, control and redirect evolution in a clinical setting.
